# STING agonist therapy in combination with PD-1 immune checkpoint blockade enhances response to carboplatin chemotherapy in high-grade serous ovarian cancer

**DOI:** 10.1038/s41416-018-0188-5

**Published:** 2018-07-26

**Authors:** Abdi Ghaffari, Nichole Peterson, Kasra Khalaj, Natasha Vitkin, Andrew Robinson, Julie-Ann Francis, Madhuri Koti

**Affiliations:** 1 Department of Biomedical and Molecular Sciences, Queen’s University, Kingston, ON Canada; 2 Department of Obstetrics and Gynecology, Kingston Health Sciences Center, Queen’s University, Kingston, ON Canada; 3 Department of Oncology, Kingston Health Sciences Center, Queen’s University, Kingston, ON Canada; 4 Cancer Biology and Genetics, Queen’s Cancer Research Institute, Queen’s University, Kingston, ON Canada

**Keywords:** Gynaecological cancer, Oncology

## Abstract

**Background:**

High-grade serous carcinoma (HGSC) of the ovary is predominantly diagnosed at late stages and primarily treated with debulking surgery followed by platinum/taxane-based chemotherapy. Although certain patients benefit significantly from currently used chemotherapy, there are patients who either do not respond or have an inadequate duration of response. We previously showed that tumours from chemoresistant patients have an immunosuppressed pre-existing tumour immune microenvironment with decreased expression of Type I Interferon (IFN1) genes.

**Methods:**

Efficacy of a ‘*ST*imulator of *IN*terferon *G*enes’ agonist was evaluated in combination with carboplatin chemotherapy and PD-1 immune checkpoint blockade therapy in the ID8-*Trp53*^−/−^ immunocompetent murine model of HGSC.

**Results:**

Treatment with STING agonist led to decreased ascites accumulation and decreased tumour burden. Survival of mice treated with a combination of carboplatin, STING agonist and anti-PD-1 antibody was the longest. Tumour immune transcriptomic profiling revealed higher IFN response, antigen presentation and MHC II genes in tumours from STING agonist-treated mice compared to vehicle controls. Flow cytometry analysis revealed significantly higher intra-tumoural PD-1^+^ and CD69^+^CD62L^−^, CD8^+^ T cells in STING agonist-treated mice.

**Conclusions:**

These findings will enable rational design of clinical trials aimed at combinatorial approaches to improve chemotherapy response and survival in HGSC patients.

## Introduction

High-grade serous carcinoma (HGSC) of the ovary is the most lethal gynaecologic malignancy leading to approximately 200,000 deaths annually across the world, with 16,000 deaths in North America each year.^1,2^ Five-year survival rates are at a dismal 45.6% for this cancer with very modest increases in the past 3 decades.^2^ The standard treatment of advanced HGSC includes cyto-reductive surgery followed by treatment with platinum and taxane-based combination chemotherapy. Most patients show an initial sensitivity to platinum, however, disease relapse occurs in over 70% of the cases.^2–4^ A major impediment to longer overall survival (OS) rates in HGSC is therefore, the development of resistance to conventional chemotherapy mostly leading to an incurable disease post-recurrence. Improvements in therapeutic strategies such as use of dose-dense chemotherapy, intra-peritoneal chemotherapy, anti-angiogenic drugs and poly-ADP-ribose polymerase I inhibitors, have shown modest increases in progression-free survival.^2^ Given the high genomic instability and immunogenic nature of HGSC tumours, several ongoing trials are evaluating the efficacy of novel immune checkpoint blockade therapies with no definitive success reported yet. Improving OS of HGSC patients therefore, needs urgent investigations on additional combinatorial approaches. Chemotherapy resistance can be intrinsic or acquired post-exposure to the drug.^5^ The most recent Gynecologic Oncology Group (GOG) classification scheme, however, defines the disease as platinum-refractory (relapse within 4 weeks of platinum therapy), resistant (relapse <6 months from platinum therapy initiation), partially platinum-sensitive (relapse within 6–12 months from platinum therapy initiation) and platinum-sensitive (relapse >12 months from platinum therapy initiation).^2^

Our previous reports on the identification of biomarkers of chemotherapy resistance in HGSC revealed that a pre-existing active T helper type I tumour immune microenvironment (TME) is associated with increased chemotherapy response, progression-free survival and OS in HGSC.^3,5,6^ These findings also confirmed the significance of a type I interferon (IFN1)-associated transcriptional profile with an enrichment of chemokine genes, *CXCL9, CXCL10* and *CXCL11* key to the recruitment of tumour infiltrating lymphocytes (TIL). Increased STAT1 expression further correlated with increased density of intra-epithelial CD8^+^ TILs suggesting the importance of their activation state in determining response.^3^ These and other similar findings confirm the importance of recruitment and activation of CD8^+^ TILs in overall prognosis and response to chemotherapy in HGSC.^7–9^ Using the ID8 syngeneic mouse model of HGSC we then demonstrated the positive impact of STAT1 induced T cell recruiting and angiostatic chemokine, CXCL10, on the TME of HGSC.^10^ Our in vivo findings supported the observation that HGSC patients presenting with lower ascites volumes have increased *CXCL10* gene expression in the corresponding tumours.^11^ These reports suggest that immune-based therapies that can reverse the immunosuppressed state of HGSC tumours to an active state via stimulating the tumour IFN1 genes, could be used to improve response to chemotherapy and patient survival rates. Therapies that stimulate IFN1 and CXCL10 production, such as toll-like receptor agonists and IFNα are currently under several trials across cancers.^12^ Poly I:C-based pre-clinical and human trials are ongoing but have not shown a benefit yet.^13^ Moreover, clinical trials using IFN alone as a therapeutic approach showed toxic side effects and local accumulation. These findings emphasise that IFN agonists stimulating endogenous IFN could prove to be more beneficial in cancer treatment. The recently discovered innate immune sensing cyclic GMP–AMP synthase (cGAS)-Stimulator of Interferon Genes (STING, encoded by *TMEM 173*) pathway is critical for cytosolic DNA sensing.^14^ Following DNA sensing, the STING pathway is activated as an early IFN1 response whereas STAT1 activation constitutes the late/chronic response.^15^ The TIL recruiting chemokine, CXCL10 is also a downstream product of endogenous STING pathway activation in addition to exogenous IFN activation. Several STING agonists are currently under development, however, the recently developed *2′3′-c-di-AM (PS) 2 (Rp, Rp)* has shown significant therapeutic benefit via enhancing dendritic cells (DC)-mediated CD8^+^ TIL recruitment to the TME in acute myeloid leukaemia, melanoma, breast and colorectal cancer models.^16–18^ Aligning with the recent classification of tumours as ‘T-cell inflamed or hot tumours’ and ‘non-T cell inflamed or cold tumours’,^19^ ours and similar findings by other groups in HGSC are compelling evidence emphasising the potential of STING agonist for enhancing chemotherapy response and OS of patients.

Given that chemoresistance is associated with cold tumours with decreased expression of IFN1 genes and lower density of CD8^+^ TILs, we hypothesised that response to chemotherapy and OS can be improved via the addition of STING agonist to the treatment regime. In the current study, we thus evaluated the efficacy of the STING agonist, *2′3′-c-di-AM (PS) 2 (Rp, Rp)*, in the ID8 syngeneic mouse model of HGSC. Indeed, ID8 tumour-bearing mice treated with STING agonist survived longer compared to vehicle control. Combination treatment with carboplatin and STING agonist showed synergistic effect and longer survival compared to monotherapy. Most importantly, mice treated with a combination of carboplatin, STING agonist and the immune checkpoint inhibiting anti-PD-1 antibody showed the longest survival compared to carboplatin + STING agonist treatment.

## Materials and methods

### Cytotoxicity and proliferation assay to evaluate the effect of STING agonist on cancer cells

The ID8-*Trp53*^*−/−*^^20^ mouse ovarian surface epithelial cells were kindly provided by Dr. Ian Mcneish (University of Glasgow). Given that mutations in *TP53* gene are present in >95% HGSC tumours, this recently developed improved ID8 cell line therefore more closely recapitulates the human HGSC tumour progression.^20^ The ID8*-Trp53*^*−/−*^ cells in Dulbecco’s Modified Eagle’s Medium (Sigma Aldrich Co. #D6429) supplemented with 2% foetal bovine serum, 100 μg/mL of penicillin/streptomycin and a solution containing 5 μg/mL of insulin, 5 μg/mL of transferrin and 5 ng/mL of sodium selenite, were seeded in 96-well plates (Sarstedt, Numbrecht, Germany) at a density of 2000 cells/well 24 h prior to treatment. Cells were then treated with increasing concentrations of STING agonist (*2′3′-c-di-AM (PS) 2 (Rp, Rp)*, Invivogen) and carboplatin (Cancer Clinic, Kingston General Hospital) or PBS, for 48 h (triplicate wells) in the presence of propidium iodide (Sigma Aldrich). Uptake of the PI fluorescent dye (cell count) and cell confluency (area covered by cells per field of view) were monitored in real-time by using live-cell imaging (IncuCyte™ Zoom, Essen Bioscience, Ann Arbor, MI). Data were normalised and presented as the percentage of control (untreated) in each cell line. Fold cytotoxicity and cell confluency at 48 h were calculated as log_10_ of drug concentration relative to control group.

### In vivo studies in the ID8-*Trp53*^−/−^ syngeneic mouse model of HGSC

**Fig. 1:**
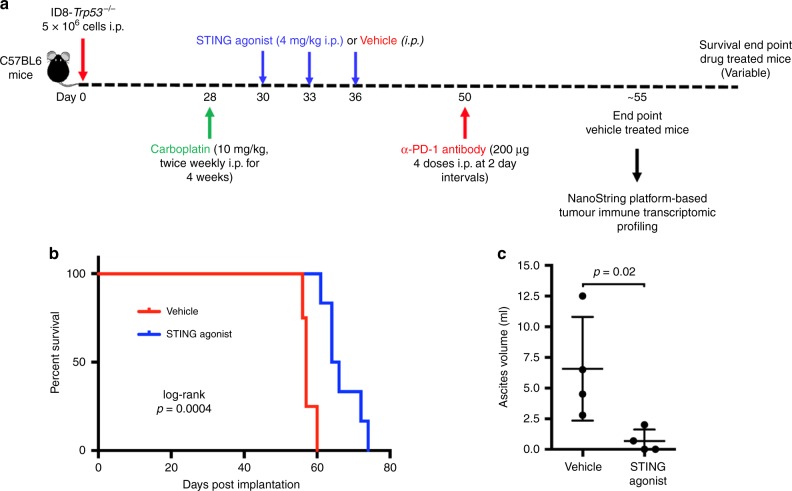
Treatment with STING agonist monotherapy increases overall survival in a mouse model of HGSC. **a** Summary of experimental design to study the effect of STING agonist in combination with carboplatin and PD-1 checkpoint inhibitor in HGSC tumours is shown. Four weeks post ID8-*Trp53*^−/−^ tumour cell injection (i.p.), mice were randomised into four treatment groups: (1) STING agonist; (2) STING agonist + carboplatin; (3) STING agonist + carboplatin + anti-PD-1 antibody; and (4) vehicle (*n* = 4, three independent experiments). All treatments were administered via i.p. route. **b** Survival analyses of tumour-bearing mice treated with STING agonist or vehicle (four mice per treatment arm, two independent experiments). Log-rank test was applied to determine statistically significant differences (*p* < 0.05) in survival. **c** Ascites volumes (mL) were measured when the vehicle mice reached endpoint (abdominal diameter of ≥35 mm; four mice per group) and compared with Mann–Whitney test

All animal protocols were approved by the Queen’s University Animal Care Committee. 5–6 × 10^6^ ID8-*Trp53*^*−/*−^ cells in 200 μl of PBS, were transplanted via intra-peritoneal injections in 8- to 10-week old female C57BL/6 mice (Charles River Laboratories International Inc). Approximately 4-weeks post tumour cell implantation, mice were randomised and treated with STING agonist alone or carboplatin + STING agonist or carboplatin + STING agonist + anti-PD-1 or vehicle via i.p. administration, at the indicated doses and time points (Fig. 1a). The anti-mouse PD-1 antibody (clone RMP1-14; BioXcell) was administered 2 weeks following the last STING agonist injection. For depletion of CD8^+^ T cells, 400 μg anti-CD8 antibody (clone 2.43; BioXcell) was administered i.p. twice weekly for first 3 weeks starting 1 day prior to STING agonist treatment initiation. Treatments with anti-CD8 depleting antibody were reduced to once a week in weeks 4 and 5.

### Mass cytometry analysis of splenocytes

To analyse immune cell proportions and phenotypic changes in the spleen, splenocytes from mice treated with carboplatin and STING agonist combination were collected at early (24 h post STING agonist administration) and mid (3 days post completion of STING agonist treatment) time points. Splenocytes were barcoded using a Cell-ID 28-Plex Maxpar mouse spleen/lymph node phenotyping panel Kit (Fluidigm) as per the manufacturer’s instructions. Additional Mouse anti-PD-L1 (153 Eu) and CD274/PD-1(159 Tb) tagged antibodies (Fluidigm) were included in the panel. Briefly, 1 × 10^7^ splenocytes/mL were labelled with Cell-ID 103 Rh (Fluidigm) and incubated for 5 min. Cell-ID 103 Rh was chosen for cell viability in lieu of Cell-ID cisplatin, due to potential for residual in vivo cisplatin interference in the Pt channel on the CyTOF. Cell surface staining was conducted by resuspension of cells at 10^6^ cells/50 ul in Maxpar Cell Staining Buffer Staining Buffer (CSB; Fluidigm) and Fc-receptor block was added. Following Fc-receptor blocking, cells were labelled via the addition of antibody cocktail mixed in CSB overnight. Labelled splenocytes were then washed twice with CSB and Cell-ID Intercalator-IR diluted in Fix and Perm buffer (Fluidigm) was added at 125 nM and incubated for 1 h. Labelled cells were washed twice in CSB and once with Milli-Q ‘ultrapure’ H_2_O (Millipore) water and centrifuged. Supernatants were discarded and residual volume of water with cells vortexed were kept in tubes prior to CyTOF data acquisition. Cell concentrations were adjusted to 5 × 10^5^ cells/mL. EQ Four Element Calibration Beads (Fluidigm) were added at a concentration of 4 × 10^4^ beads/mL. Cells were filtered and analysed on a Helios mass cytometer (CyTOF; Fluidigm). Parameters of 0.030 mL/min, total event threshold of 100,000, 90 s acquisition delay and 10 s detector stability delay, were used for all samples. CyTOF Data were exported as FCS files and normalised with calibration bead readings from the manufacturer’s software (version 6.0.626). Normalised files were deconvoluted using Fluidigm Debarcoder software. All gating and analysis was conducted using FlowJo v10 software (FlowJo LLC, USA). Gating strategy was performed as per Fluidigm MaxPar Mouse Spleen/Lymph Node Phenotyping panel kit (Fluidigm, USA). Statistical analysis of CyTOF data was performed using GraphPad Prism 7.03 (GraphPad, USA). Briefly, time point groups were sorted by time point (early and mid). Differences between immune cell proportions at early and mid-time points from carboplatin + vehicle and carboplatin + STING agonist-treated mice were analysed using a Student’s *t*-test. *p* < 0.05 was considered statistically significant.

### Flow cytometry analysis of dissociated tumours

All mice from STING agonist-treated group and vehicle-treated group were sacrificed when vehicle-treated mice reached endpoint. Following euthanasia, ascites fluid was aspirated using 18-gauge needle. Tumours were excised, minced, and digested in RPMI-1640 media containing 2% FBS (Sigma-Aldrich), 1 mg/mL collagenase IV (Sigma-Aldrich), and 20 µg/mL DNase (Sigma-Aldrich) with gentle continuous agitation. After 60 min digestion at 37 °C, cells were passed through a 70-µm filter, washed by PBS-EDTA, and placed in FACS PAB buffer (PBS + 0.5% BSA). Single-cell suspensions were then analysed by flow cytometry using a Beckton Dickenson FACS Aria III (BD Biosciences, Mississauga, ON) and FITC-, phycoerythrin (PE)-, PE/Cy5-, PE/Cy7-, allophycocyanin (APC)-, APC/Cy7-, and Alexa488-conjugated antibodies against CD4, CD8b, CD11c, CD103, CD69, CD62L, and PD-1 cell surface markers. Matched isotype controls were used for each antibody to determine the gates.

### Multiplex cytokine analysis from plasma

Plasma samples collected at three different time points (early and late) as indicated, post STING agonist treatment were subject to multiplexed cytokine profiling using the MD31, 31-plex cytokine/chemokine array (Eotaxin, G-CSF, GM-CSF, IFN gamma, IL-1alpha, IL-1beta, IL-2, IL-3, IL-4, IL-5, IL-6, IL-7, IL-9, IL-10, IL-12 (p40), IL-12 (p70), IL-13, IL-15, IL-17A, IP-10, CXCL1, LIF, LIX, MCP-1, M-CSF, MIG, MIP-1alpha, MIP-1beta, MIP-2, RANTES, TNF alpha, VEGF) at Eve Technologies Corporation (Calgary, AB, Canada).^10^ All samples were analysed in triplicates. The standard curve regression was used to calculate the concentration of each target cytokine. Data was analysed using GraphPad Prism (7.02).

### Tumour immune transcriptomic profiling using NanoString-based gene expression profiling

To determine the effect of STING agonist treatment on the TME, total RNA was isolated from fresh frozen tumour tissues collected from STING agonist-treated and vehicle-treated mice, at endpoint using the total RNA Purification Kit (Norgen Biotek Corporation) as per the manufacturer’s instructions. RNA concentration and purity was estimated on a NanoDrop ND-100 spectrophotometer (NanoDrop Technologies, Wilmington, DE, USA). 100 ng of total RNA from each tumour sample was subjected to digital multiplexed profiling, using the pre-built nCounter Mouse PanCancer Immune Profiling panel (700 mouse immune-related genes with 40 housekeeping controls, NanoString Technologies Inc.) as per our previously established protocols.^5,10^ Normalisation of raw data was performed using the nSolver software 3.0 (NanoString Technologies, Seattle, WA). The raw NanoString counts were initially subjected to normalisation for all target RNAs in all samples based on built-in positive controls. This step accounts for inter-sample and experimental variation such as hybridisation efficiency and post-hybridisation processing. The geometric mean of each control was calculated to indicate the overall assay efficiency. The housekeeping genes were used for mRNA content normalisation. Differentially expressed genes between the tumours from STING agonist-treated and vehicle-treated mice were derived using GraphPad Prism software. A *p*-value < 0.05 was considered statistically significant.

## Results

### Treatment with STING agonist improves survival, reduces ascites formation and tumour burden in the ID8*-Trp53*^−/−^ mouse model of HGSC

Previous reports on the STING agonist used in our study have demonstrated its efficacy via administration by intra-tumoural route. Given the peritoneal dissemination of ovarian tumours, we first evaluated the efficacy of STING agonist monotherapy administered via i.p. route. To compare the effect of STING agonist monotherapy on ascites accumulation and tumour burden in comparison with vehicle-treated mice, the STING agonist-treated mice were sacrificed when the vehicle mice reached endpoint (abdominal diameter of ≥35 mm). Mice treated with STING agonist only had a significantly longer median OS of 65 days compared to the vehicle-treated mice in which the median OS was 56 days (Fig. 1b, *p* = 0.0004). Significantly decreased ascites volumes and lower tumour burden was observed in the STING agonist-treated mice (average 0.68 mL) compared to vehicle-treated mice (average 6.5 mL, Fig. 1c). STING agonist revealed no significant effect on ID8-*Trp53*^*−/−*^ cells proliferation or cell death in vitro (Figure S1, supplementary figure). These results suggest the potential therapeutic benefit of the addition of STING agonist treatment in HGSC.

### Treatment with STING agonist following carboplatin leads to splenic population changes and PD-1/PD-L1 immune checkpoint expression

**Fig. 2:**
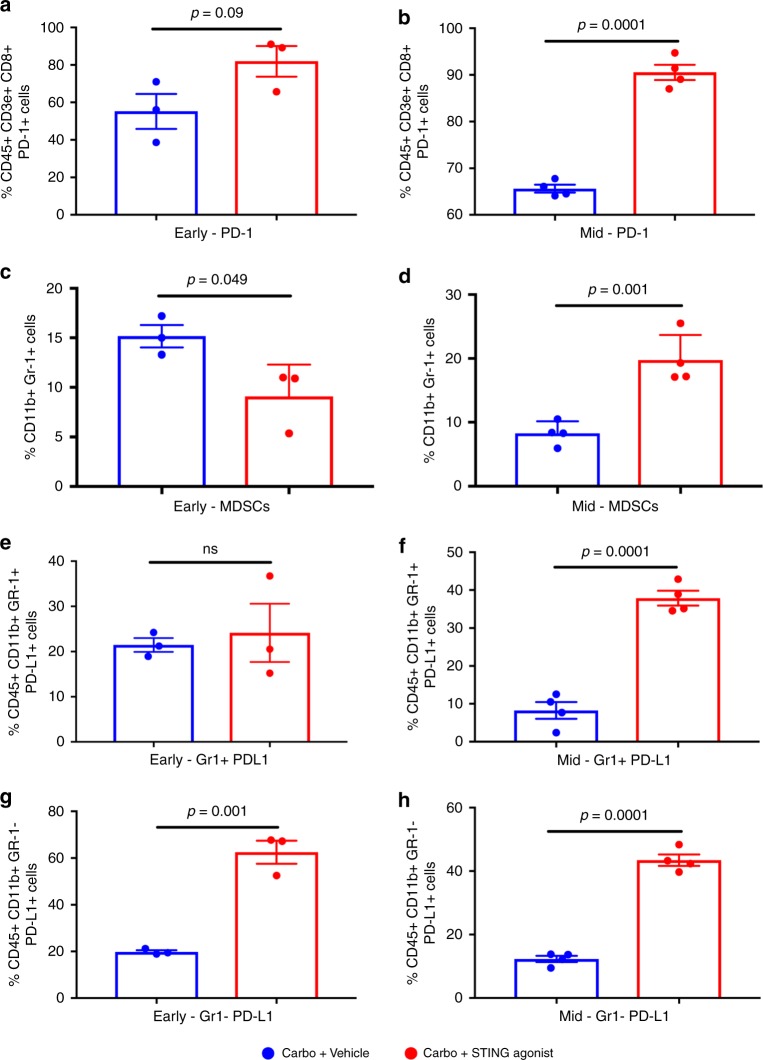
Carboplatin + STING agonist combination treatment leads to increased splenic MDSCs and immune checkpoint expression in splenocytes. Single cells suspensions of splenocytes collected at early (24 h) and mid (10 days) time points post initiation of STING agonist treatment, were subjected to immune cell phenotyping using CyTOF-based mass cytometry. Significant increases in PD-1^+^ CD8^+^ cytotoxic T cells (**b**), in MDSCs (**d**), CD11b^+^Gr1^+^PD-L1^+^ MDSCs (**f**), and CD11b^+^ PD-L1^+^ macrophages (**g**, **h**) were observed in carboplatin + STING agonist-treated mice compared to the carboplatin + vehicle controls at mid-time points. All gating and analysis was conducted using FlowJo v10 software (FlowJo LLC, USA). Gating strategy was performed as per Fluidigm MaxPar Mouse Spleen/Lymph Node Phenotyping panel kit (Fluidigm, USA). Statistical analysis of CyTOF data was performed using GraphPad Prism 7.03 (GraphPad, USA)

To investigate the effect of STING agonist treatment in combination with carboplatin on splenic immune cell phenotypic changes post-treatment, we used CyTOF mass cytometry-based immune cell analysis at early and mid-time points in mice treated with carboplatin + vehicle or carboplatin + STING agonist. We observed significantly higher splenic PD-1^+^CD8^+^ T cells only at mid-time point in the carboplatin + STING agonist-treated group (Fig. 2a, b). Interestingly, at early time point, the carboplatin + vehicle-treated group showed significantly increased levels of myeloid-derived suppressor cells (MDSCs; Fig. 2c) whereas, these changes were reversed at mid-time point, with significantly high MDSCs in the carboplatin + STING agonist-treated group (Fig. 2d). Further, significant increases in splenic PD-L1^+^ CD11b^+^ Gr-1^+^ MDSCs were observed in the carboplatin + STING agonist-treated mice compared to the carboplatin + vehicle-treated mice only at mid-time point (Fig. 2e, f). At both early and mid-time points post-treatment, significant increases in splenic CD11b^+^ PD-L1^+^ macrophages were also observed in the carboplatin + STING agonist-treated mice compared to carboplatin + vehicle-treated mice (Fig. 2g, h). These data confirm that effects of STING agonist on immune checkpoint expression on splenic myeloid cells and thus provide the rationale for treatment with PD-1/PD-L1 immune checkpoint blockade post STING agonist treatment.

### Treatment with STING agonist modulates the TME towards a T_H_1 type response

**Fig. 3:**
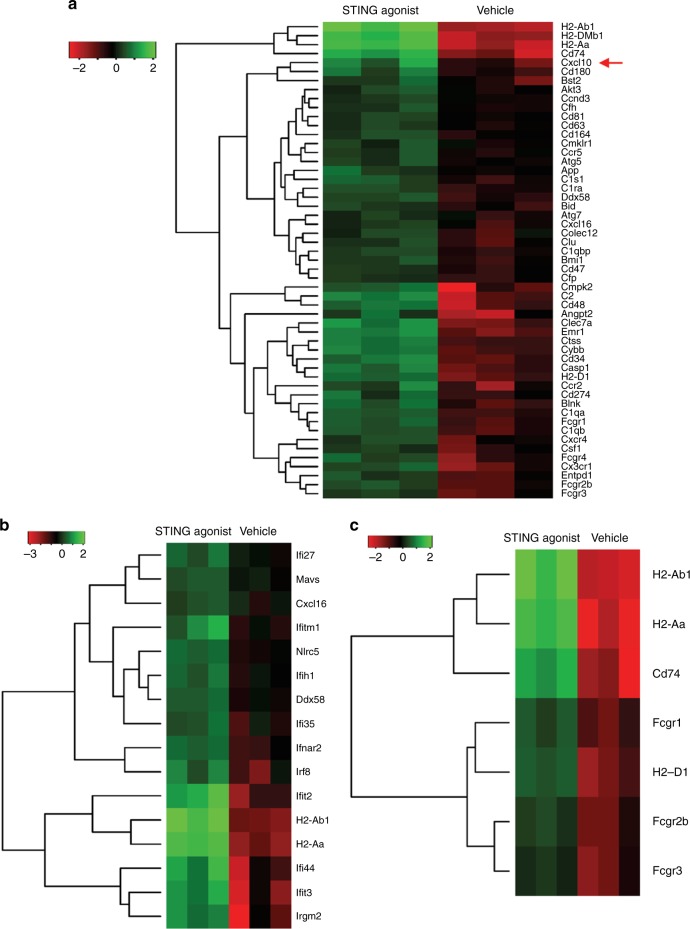
STING agonist treatment enhances a T_H_1 type response in TME. NanoString-based tumour immune transcriptome profiling showing **a** significantly differentially expressed immune response, **b** IFN pathway, and **c** antigen processing and presentation pathway associated genes in ID8-*Trp53*^−/−^ tumours collected at endpoint from STING agonist (i.p.) and vehicle-treated mice (*n* = 3 each group)

**Fig. 4:**
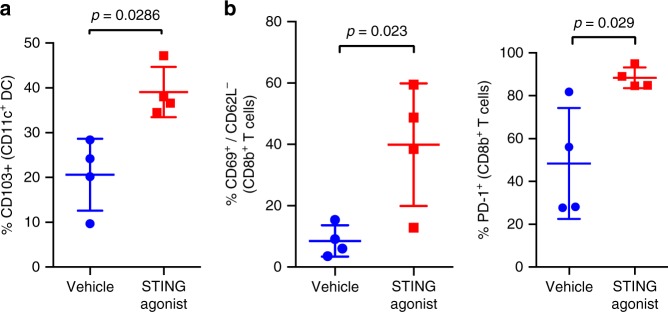
Treatment with STING agonist increases CD103^+^ DC and activated CD8^+^ T cell infiltration in the TME of a mouse model of HGSC. Flow cytometry analysis of ID8-*Trp53*^−/−^ tumours treated with vehicle or STING agonist (day 55 after tumour injection and day 14 post STING agonist treatment) showing subset of CD103^+^ DCs (Panel **a**, CD11c^+^ gate) and CD69^+^/CD62L^−^ and PD-1^+^ TILs (Panel **b**, CD8^+^ gated). Mann–Whitney test was used to compare statistically significant differences between vehicle and STING agonist groups

To evaluate the effect of STING agonist treatment on alterations in tumour immune profiles, we subjected the total RNA from tumours in the STING agonist only and vehicle-treated mice to NanoString-based immune transcriptomic profiling. A total of 141 differentially expressed genes (fold change difference >1.5, *p* < 0.05) were observed between the STING agonist-treated tumours compared to the vehicle-treated tumours (Supplementary Table 1). Overall, an enrichment of genes associated with antigen presentation, MHC II (such as *H2-Ab1, H2-DMb1, H2-Aa* and *Cd74*) and interferon response (*Ddx58, Ifr8, Ifit2, Ifi44, Ifit3* and others) was seen in tumours from the STING agonist-treated mice (Fig. 3a–c) compared to the vehicle-treated mice. Most importantly significantly increased expression of *Stat1* (6.6-fold increase) and *Cxcl10* (3.5-fold increase) were noted in the tumours from STING agonist-treated mice. Flow cytometry revealed significantly higher CD103^+^CD11c^+^ DCs (Fig. 4a) and elevated PD-1^+^ and CD69^+^CD62L^−^CD8^+^ TILs (Fig. 4b), in the tumours from STING agonist-treated mice. These findings, although do not establish the primary effector cell types mediating the effects of STING agonist treatment, confirm that STING agonist treatment enhances antigen processing and presentation in the DCs in the TME. An increased activation of IFN pathways leading to *Cxcl10* expression then putatively leads to increased influx and cross-priming of CD8^+^ TILs in the TME.

### STING agonist treatment induced systemic pro-inflammatory cytokine levels are independent of CD8^+^ T cells

**Fig. 5:**
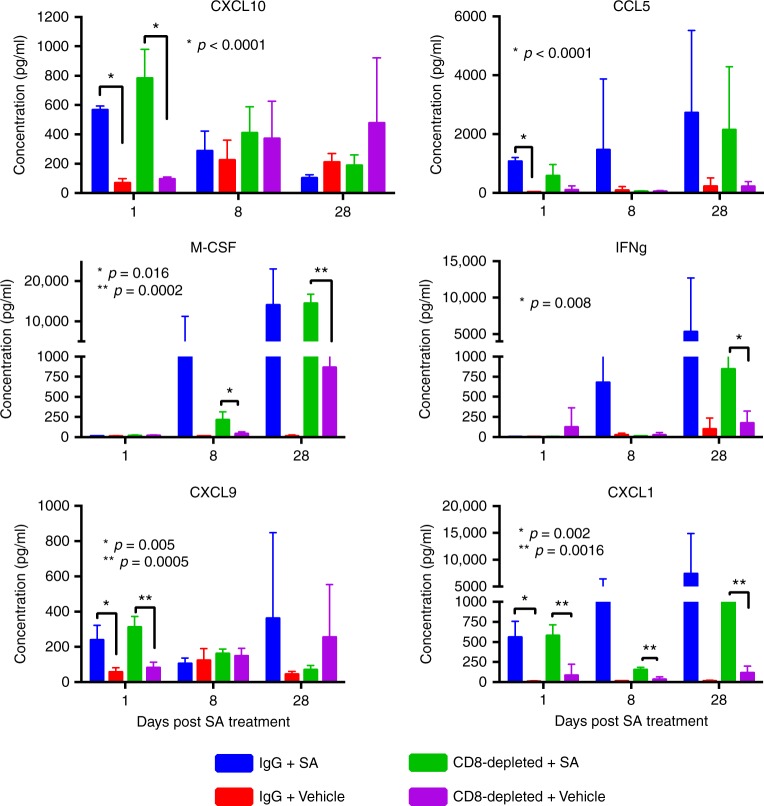
STING agonist treatment induces pro-inflammatory cytokine levels independent of CD8^+^ T cells. Plasma inflammatory cytokines levels (pg/mL) in ID8-*Trp53*^−/−^ tumour-bearing mice treated with isotype control (IgG) or anti-CD8 antibody, at day 1, 8, and 28 post-treatment with STING agonist or vehicle. Plasma samples on days 1, 8, and 28 post STING or vehicle treatment were collected from separate groups of mice following α-CD8 antibody-mediated T cell depletion (injections started 1 day prior to STING agonist treatment). Data are shown as mean ± SEM of at least four mice per group. Mann–Whitney *t*-test was used for comparison between vehicle and treated samples

We first established that treatment with STING agonist led to reduced tumour burden and significantly reduced ascites volumes compared to vehicle group suggesting a localised effect of STING agonist on tumour growth and survival. We then asked whether the systemic immune response could be a measure of the effects of STING agonist. In order to determine the effect of STING agonist treatment on circulating pro-inflammatory cytokine profiles, we evaluated plasma cytokines in tumour-bearing mice at days 1, 8, and 28 post STING agonist treatment initiation, using a pre-built cytokine panel representing primary mediators of inflammation. In the STING agonist-treated tumour-bearing mice, we found significantly elevated levels of the cytokines, CXCL10, CCL5, IFN-γ, M-CSF, CXCL9 and CXCL1 at early, mid and late time points in comparison to levels in plasma from vehicle-treated mice (Fig. 5). This observation is consistent with the expected effect of STING agonist treatment in activating various immune cell types and the resultant cytokine production.

Given the previous reports on STING agonist function through CD8^+^ T cell cross-priming, and to confirm if the effect of STING agonist is entirely dependent of CD8^+^ TILs, we also aimed to evaluate the impact of STING agonist in the absence of CD8^+^ T cells. Moreover, given the pre-treatment TME states with reduced CD8^+^ TIL density in cold tumours or those exhibiting T cell exclusion, it is necessary to evaluate whether STING agonist treatment could reverse these to a T cell inflamed state. In the predominant lymphocyte-derived cytokine context, CXCL9, CXCL10 (produced by lymphocytes, epithelial cells and involved in lymphocyte recruitment) and CCL5 (produced by variety of immune cells) were found to be increased at days 1 and 8 post-treatment even in the CD8^+^ T cell depleted mice that were treated with STING agonist compared to the isotype control + STING agonist group (Fig. 5). An important observation in this study was the significantly higher levels of IFN-γ seen in the CD8^+^ T cell depleted + STING agonist group at 28 days post-treatment which indicates a potential role of natural killer (NK) cells, another source of IFN-γ, in mediating STING agonist effects. Indeed, higher M-CSF levels also indicate the myeloid cell expansion. In summary, pro-inflammatory plasma cytokine profiles at early, mid and late time points post-treatment, reflect the prolonged effects of STING agonist and can act as markers of response post-treatment.

### STING agonist treatment shows synergistic effect with carboplatin chemotherapy and PD-1 immune checkpoint blockade therapy

**Fig. 6:**
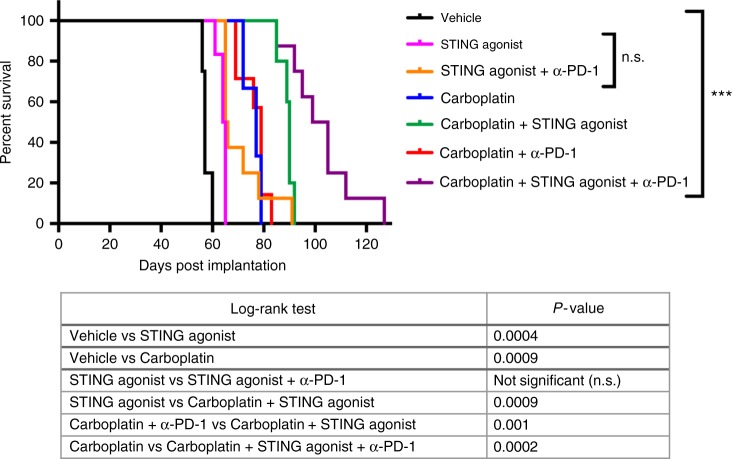
STING agonist enhances response to chemotherapy in a mouse model of HGSC. Kaplan–Meier survival analysis showing significantly increased survival of mice treated with carboplatin + STING agonist + anti-PD-1 or carboplatin + STING agonist combination compared to carboplatin only, STING agonist only and vehicle-treated mice. Log-rank (Mantel Cox) test was applied to derive significant differences (*p* < 0.05) in survival between different treatment groups (Table). For each treatment arm, 6–8 mice were included

Platinum is used as first-line chemotherapy in HGSC treatment. Therefore, following evaluation of the efficacy of STING agonist monotherapy and its impact on the TME, we tested the effect of combining STING agonist with carboplatin on survival. Based on the results from our flow cytometry analysis that showed higher PD-1^+^CD8^+^ TILs in STING agonist-treated tumours, we added anti-PD-1 antibody treatment to one arm of the study where mice were treated with a combination of carboplatin and STING agonist. Kaplan–Meier survival analysis and log-rank (Mantel-Cox) test showed significant differences in the survival of mice following the addition of STING agonist. Mice treated with a combination of carboplatin, STING agonist and anti-PD-1 antibody showed the longest survival followed by carboplatin and STING agonist group, carboplatin only, STING agonist only and vehicle (Fig. 6). Importantly, mice treated with carboplatin + STING agonist showed significantly longer survival compared to the carboplatin + anti-PD-1 treated group. No significant differences between survival of STING agonist-treated mice compared STING agonist + anti-PD-1 treated mice were observed. No toxicity was observed with the dose of STING agonist used in all treatment combinations. All treated mice were apparently healthy with no signs of distress post-treatment. These results provide the rationale for the addition of STING agonist to the treatment regime following carboplatin treatment initiation. The significantly longer survival observed when PD-1 antibody treatment was added to the combination with carboplatin and STING agonist-treated mice, provides pre-clinical evidence for its optimal sequencing in human HGSC treatment.

## Discussion

We previously showed that intrinsically chemoresistant HGSC tumours, exhibit an immunologically cold TME with a reduced density of CD8^+^ TILs and that the decreased IFN1 gene expression in the cold tumours is reflective of poor anti-tumour immune responses.^3,5^ Using the in vivo ID8-*Trp53*^−*/−*^ syngeneic mouse model of HGSC, we also reported the positive impact of IFN induced TIL recruiting chemokine, CXCL10, in reducing tumour burden and enhancing IFN1 immune responses in the TME.^10^ Enhancing anti-tumour TIL recruitment via CXCL10 induction is thus a promising approach to improve chemotherapy response and OS of HGSC patients. The current study was conducted with an aim to evaluate the effect of STING agonist as a combinatorial immunomodulatory agent for HGSC treatment. We chose the recently developed STING agonist that has been previously shown to stimulate IFN genes in immune cells and convert cold tumours to immunologically hot tumours in various models of solid tumours including breast, colorectal, melanoma and acute myeloid leukaemia.^14,21^ Given that peritoneal carcinomatosis is exhibited by most HGSC patients presenting with advanced stage disease, we tested the effect of STING agonist, delivered via i.p. route, on the TME profile and OS. Our finding that treatment with STING agonist monotherapy led to significantly reduced ascites accumulation, decreased tumour burden and increased OS compared to vehicle-treated controls establishes, for the first time, the promising therapeutic potential of immune stimulatory STING agonist in HGSC. Immune transcriptomic analysis of tumours from STING agonist-treated mice showed an increased expression of genes involved in MHC II/antigen processing and presentation and active IFN response pathways. Significantly increased transcript levels of IFN induced genes such as *Stat1* and *Cxcl10* indicate the activation of IFN pathways in the TME by STING agonist treatment leading to downstream protective effects. These findings are in concordance with previous reports on this agent in other models of solid tumours. Abdominal diameter is an established and accurate surrogate of endpoint in the ID8 murine model of HGSC. The most important finding in our study was that the mice treated with STING agonist showed significantly reduced ascites and decreased tumour burden compared to vehicle-treated mice. Reduced ascites accumulation could potentially result from the angiostatic effects of CXCL10, a chemokine that is induced and released in the TME post-IFN pathway activation via both STING/STAT1 or could also be due to decreased tumour burden. This notion is supported by our recent report where we show the positive impact of high CXCL10 levels in the TME.^10^

A key clinically important finding from our study is the observation that mice treated with carboplatin and STING agonist showed significantly longer survival compared to carboplatin alone. This effect could be mediated by immunogenic cell death (ICD) resulting from carboplatin treatment, which further enhanced the response to STING agonist via amplifying the IFN response in the antigen presenting cells that respond to the chemotherapy-induced danger-associated molecular patterns released upon cellular damage.^22^ The ICD inducing ability of carboplatin has not been fully established and thus we speculate that treatment with anthracyclines such as doxorubicin, which is a potent ICD inducer could prove more beneficial. However, it is also possible that the longer survival of carboplatin + STING agonist-treated mice compared to either alone, could result from the synergistic effect of the combination treatment. The finding that STING agonist treatment alone increased OS, significantly delayed ascites accumulation and reduced tumour burden indicates its anti-tumour roles, which needs further mechanistic studies. Indeed, our in vitro studies confirmed that STING agonist does not lead to direct killing of cancer cells, which suggests the role of immune cells such as macrophages, DCs, NK cells or CD8^+^ TILs.

Analysis of immune cell proportions in splenocytes at early and mid-time points post STING agonist treatment revealed changes appearing at early time point with significant differences at mid-time point or end of STING agonist treatment. An important finding relevant to potential future combination immune checkpoint treatment approaches was the significantly increased splenic MDSCs and PD-L1^+^ macrophages and MDSCs. These results indicate activation of interferon-stimulated genes leading to high PD-L1 levels in the myeloid cells post STING agonist treatment. Simultaneously increased levels of CD8^+^PD-1^+^ T cells in spleen also suggest that early activation of CD8+ T cells could increase IFNγ levels that induces PD-L1 expression on myeloid cells. Other sources of IFNγ such as NK cells could also exist although we did not observe significant changes in these population. Endogenous STING pathway activation in antigen presenting cells leads to PD-L1 expression,^23,24^ which could be another possible mechanism leading to high PD-L1 levels in splenocytes. Increased CD69^+^CD62L^+^ and PD-1^+^CD8 T cells in the tumours and splenocytes of STING agonist-treated mice was indicative of increased infiltration by both activated and exhausted cytotoxic T cell phenotypes. Furthermore, the addition of STING agonist led to superior survival compared to the addition of anti-PD-1 to the carboplatin treatment, which suggests the critical need for immune priming.

We thus added the anti-PD-1 antibody to the treatment regime following carboplatin + STING agonist treatment. As expected, we observed the longest survival in mice treated with carboplatin + STING agonist + PD-1 antibody followed by carboplatin + STING agonist followed by carboplatin only and STING agonist only groups. Although survival benefit is achieved with this combinatorial regime, we observed an eventual relapse (although delayed) and ascites formation. This finding leads us to the hypothesis that increased systemic and local IFN-γ resulting from activation of CD8^+^ TILs post STING agonist treatment could induce increased expression of immunosuppressive factors such as PD-L1 and/or IDO1 on cancer cells and/or immune cells, mainly on activated myeloid cells including macrophages, DCs and MDSCs.^24,25^ Such effects are classified as acquired or adaptive immune resistance in the TME that result in exhaustion and evasion of an adaptive anti-tumour immune response.^26,27^ This finding is not only key to the ongoing trials targeting the PD-1/PD-L1 axis in ovarian cancer but also provides the basis for the sequencing of STING agonist administration post chemotherapy to improve patient outcomes.

As reported by Spitzer et al., in addition to localised anti-tumour immune responses, a broader systemic analysis is required to evaluate the efficacy of immunotherapies in cancer.^28^ We speculate that the source of increased levels of IFN-γ, M-CSF and CXCL1 in CD8^+^ T cell depleted mice treated with STING agonist could potentially be NK cells or antigen presenting cells or other myeloid-derived cells. Overall, our systemic cytokine data from tumour-bearing mice treated with or without STING agonist in the presence or absence of CD8^+^ T cells shows that the effect of STING agonist treatment is not restricted to the localised tumour microenvironment. Indeed, an abscopal effect of STING agonist treatment has been previously reported.^18,29,30^ While it is an indirect evidence suggesting immunomodulatory role of STING agonist, our data from systemic cytokine analysis further explain the beneficial effects of STING agonist treatment in reducing tumour burden in STING agonist-treated mice. A potential logical extension could be the addition of the second regime of STING agonist treatment given the result that mice treated with STING agonist survived only close to 2 additional weeks compared to the vehicle control. Findings from our study strongly suggest that HGSC patients can significantly benefit from treatment with STING agonist to enhance anti-tumour immune response or overcome adaptive immune resistance, via enhanced CD8^+^ TIL cross-priming post chemotherapy, when administered selectively in patients with an under-reactive TME. Since ovarian tumours responded poorly to immune checkpoint blockade, findings from our will be foundational to the design of treatment strategies that can potentially combine STING agonist to enhance response. Given the high prevalence of defects in DNA damage repair pathways, in 50% of HGSC tumours, future studies should evaluate the efficacy of STING agonist as a combinatorial immunotherapeutic agent in tumours with defects in DNA damage such that excessive IFN activation and a state of tolerance is avoided. Overall, the current study establishes for the first time the pre-clinical basis of the potential of STING agonist as a combinatorial immunomodulatory agent that can enhance response to chemotherapy in HGSC patients.

## Electronic supplementary material

Figure S1 - STING agonist treatment did not lead to cytotoxic effect in ID8-Trp53-/- cells in vitro

Supplementary Figure legend

Supplementary Table
