# Supplementary figures and images for: STING agonist therapy in combination with PD-1 immune checkpoint blockade enhances response to carboplatin chemotherapy in high-grade serous ovarian cancer
Source: Br J Cancer. 2018 Jul 26;119(4):440–9. doi: 10.1038/s41416-018-0188-5 (PMC6133940; doi:10.1038/s41416-018-0188-5)

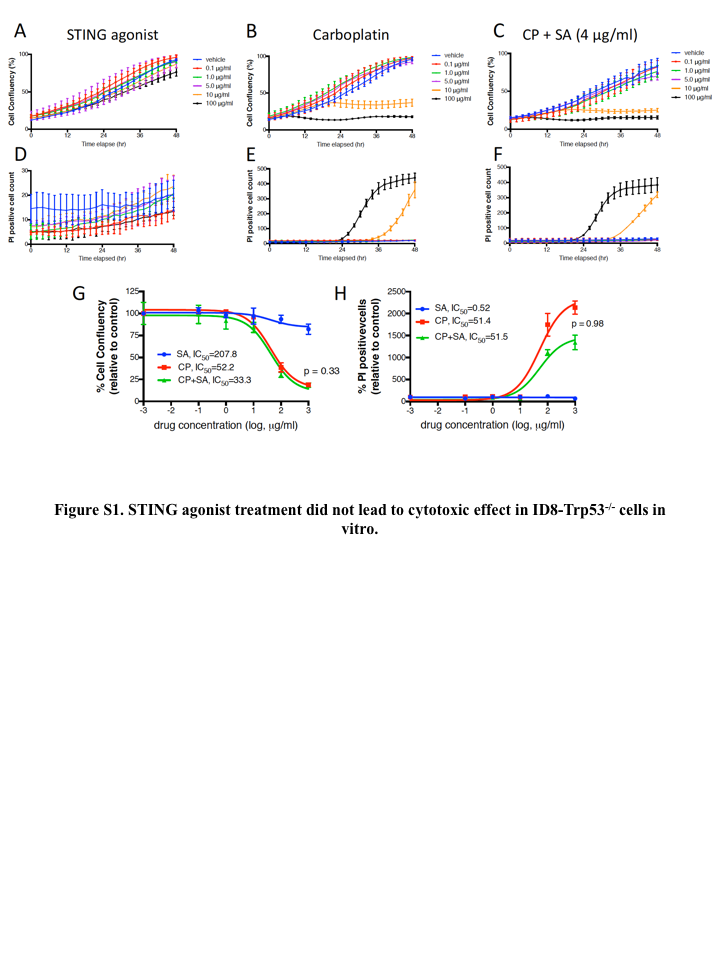

Supplement: Supplementary file 1 — Figure S1 - STING agonist treatment did not lead to cytotoxic effect in ID8-Trp53-/- cells in vitro [file 41416_2018_188_MOESM1_ESM.tif]
