# Supplementary material for: STING agonist therapy in combination with PD-1 immune checkpoint blockade enhances response to carboplatin chemotherapy in high-grade serous ovarian cancer
Source: Br J Cancer. 2018 Jul 26;119(4):440–9. doi: 10.1038/s41416-018-0188-5 (PMC6133940; doi:10.1038/s41416-018-0188-5)
Supplement: Supplementary file 2 — Supplementary Figure legend [file 41416_2018_188_MOESM2_ESM.docx]

**Supplementary figures**

**Figure S1. STING agonist revealed no cytotoxic effect in ID8-*Trp53-/-* cell line in vitro**.

ID8-*Trp53^-/-^* cells (2,000/well) were treated with increasing dose of STING agonist (panels A, D), Carboplatin (CP; panels B, E), and combination of fixed STING agonist (4 μg/ml) with increasing dose of Carboplatin (panels C, F) in DMEM media containing propidium iodide (PI) dye to identify dead cell nuclei. Cells were imaged (4 per well) every 2 hours for 48 hours on the Essen BioScience IncuCyte. Cell proliferation was indirectly assessed by measuring percent confluency (area covered by cells / total area of field of view) (panels A, B, and C). Cell death was measured by number of PI-positive cells per image (panels D, E, and F). Following log transformation of data at 48hr time point, IC50 values for STING agonist in ID8-*Trp53^-/-^* cells were calculated by nonlinear regression (least square fit) analysis in prism (panels G, H).
