# Supplementary material for: STING agonist therapy in combination with PD-1 immune checkpoint blockade enhances response to carboplatin chemotherapy in high-grade serous ovarian cancer
Source: Br J Cancer. 2018 Jul 26;119(4):440–9. doi: 10.1038/s41416-018-0188-5 (PMC6133940; doi:10.1038/s41416-018-0188-5)
Supplement: Supplementary file 3 — Supplementary Table [file 41416_2018_188_MOESM3_ESM.docx]

**Supplementary Table 1.** List of significantly differentially expressed genes in tumours from STING agonist treated mice compared to tumours from vehicle treated mice.

| **Gene name** | **Fold change** | **p-value** |
| --- | --- | --- |
| *H2-Ab1* | 13.15 | 8.22E-04 |
| *H2-Aa* | 12.15 | 7.18E-05 |
| *H2-Eb1* | 10.68 | 1.51E-04 |
| *H2-DMb1* | 10.10 | 1.78E-04 |
| *H2-Dma* | 9.89 | 4.58E-05 |
| *Cd74* | 7.54 | 3.52E-04 |
| *Ifit2* | 7.16 | 8.70E-03 |
| *Stat1* | 6.61 | 4.87E-03 |
| *Ifit3* | 6.02 | 4.41E-03 |
| *Ifi44* | 5.75 | 1.24E-02 |
| *Ly86* | 5.64 | 1.77E-04 |
| *Herc6* | 5.34 | 7.15E-03 |
| *Irgm2* | 4.81 | 2.91E-03 |
| *Clec7a* | 4.64 | 1.27E-03 |
| *C2* | 4.57 | 3.16E-04 |
| *Emr1* | 4.40 | 1.70E-04 |
| *Selplg* | 4.25 | 1.47E-04 |
| *Isg15* | 4.22 | 9.83E-03 |
| *Rps6* | 4.19 | 1.94E-05 |
| *Psmb8* | 4.07 | 2.69E-03 |
| *Cd48* | 3.90 | 6.36E-04 |
| *Stat2* | 3.66 | 3.33E-03 |
| *Xaf1* | 3.64 | 4.65E-03 |
| *Cmpk2* | 3.63 | 3.10E-03 |
| *Cxcl10* | 3.57 | 1.56E-02 |
| *Cd34* | 3.51 | 2.06E-03 |
| *Kdr* | 3.50 | 1.32E-02 |
| *Cybb* | 3.45 | 2.58E-04 |
| *H2-D1* | 3.44 | 1.29E-04 |
| *Casp1* | 3.43 | 1.09E-03 |
| *Nrp1* | 3.29 | 1.52E-03 |
| *Ctss* | 3.27 | 1.12E-04 |
| *Psmb10* | 3.27 | 2.24E-03 |
| *Irf8* | 3.20 | 1.28E-02 |
| *Ifitm1* | 3.17 | 2.62E-02 |
| *Tap1* | 3.10 | 2.58E-03 |
| *Angpt2* | 3.07 | 2.80E-02 |
| *Pecam1* | 2.95 | 6.09E-03 |
| *H2-K1* | 2.90 | 1.64E-04 |
| *Cx3cr1* | 2.89 | 4.61E-03 |
| *Ifnar2* | 2.83 | 6.40E-04 |
| *Ccr2* | 2.76 | 3.41E-02 |
| *Blnk* | 2.74 | 1.51E-03 |
| *Mef2c* | 2.72 | 8.17E-03 |
| *Psmb9* | 2.66 | 2.32E-03 |
| *H2-M3* | 2.66 | 8.10E-03 |
| *Il1rap* | 2.65 | 4.79E-02 |
| *Thbd* | 2.63 | 6.43E-03 |
| *Cd180* | 2.60 | 9.02E-03 |
| *Fcgr1* | 2.58 | 7.79E-04 |
| *H2-T23* | 2.53 | 1.07E-03 |
| *Fcgr4* | 2.52 | 8.68E-03 |
| *C1qa* | 2.51 | 1.13E-04 |
| *Thy1* | 2.50 | 2.71E-02 |
| *Hmgb1* | 2.48 | 1.65E-03 |
| *C1qb* | 2.46 | 3.99E-04 |
| *Ddx60* | 2.42 | 3.23E-02 |
| *Tlr4* | 2.36 | 7.16E-03 |
| *Psmb7* | 2.36 | 5.06E-03 |
| *Tnfsf12* | 2.36 | 4.55E-02 |
| *Nlrc5* | 2.36 | 2.58E-04 |
| *St6gal1* | 2.36 | 1.39E-04 |
| *Fcgr2b* | 2.34 | 2.47E-03 |
| *Myd88* | 2.32 | 1.03E-02 |
| *Lbp* | 2.30 | 4.78E-04 |
| *Cd274* | 2.27 | 2.08E-02 |
| *Ifi35* | 2.27 | 1.19E-02 |
| *Oas2* | 2.26 | 1.24E-02 |
| *Mr1* | 2.18 | 3.88E-02 |
| *Entpd1* | 2.17 | 8.18E-03 |
| *C1s1* | 2.14 | 3.90E-03 |
| *Fcgr3* | 2.14 | 7.52E-03 |
| *Mapkapk2* | 2.14 | 4.73E-03 |
| *Ifih1* | 2.14 | 7.32E-03 |
| *Bst2* | 2.13 | 2.45E-02 |
| *Ddx58* | 2.10 | 8.05E-04 |
| *Runx1* | 2.10 | 3.41E-03 |
| *Psen1* | 2.10 | 5.96E-03 |
| *Lcp1* | 2.07 | 1.87E-02 |
| *Socs1* | 2.06 | 4.06E-02 |
| *Pik3cd* | 2.05 | 6.97E-03 |
| *Cxcr4* | 2.02 | 1.28E-02 |
| *Irf1* | 2.01 | 1.63E-03 |
| *C1ra* | 1.99 | 5.78E-04 |
| *Ptprc* | 1.99 | 1.57E-02 |
| *Clu* | 1.96 | 1.10E-02 |
| *Itgb2* | 1.94 | 2.14E-03 |
| *Csf1* | 1.91 | 1.66E-02 |
| *Ifi27* | 1.89 | 1.12E-02 |
| *Bmi1* | 1.89 | 5.92E-03 |
| *C1qbp* | 1.88 | 1.41E-03 |
| *Tgfb2* | 1.88 | 3.76E-02 |
| *Tcf7* | 1.88 | 2.56E-03 |
| *Tmed1* | 1.87 | 4.01E-03 |
| *Bid* | 1.86 | 4.10E-03 |
| *Lamp2* | 1.86 | 1.23E-02 |
| *Litaf* | 1.86 | 7.91E-03 |
| *Tapbp* | 1.83 | 1.22E-03 |
| *Colec12* | 1.80 | 3.73E-02 |
| *Il13ra1* | 1.77 | 1.21E-02 |
| *Icosl* | 1.76 | 2.14E-02 |
| *Ubc* | 1.74 | 2.45E-03 |
| *Cd164* | 1.71 | 9.53E-03 |
| *Cfp* | 1.71 | 9.17E-03 |
| *Map2k1* | 1.71 | 3.32E-02 |
| *App* | 1.70 | 3.99E-02 |
| *Stat5b* | 1.70 | 1.87E-02 |
| *Pml* | 1.69 | 2.74E-03 |
| *Cd47* | 1.68 | 4.59E-03 |
| *Jak2* | 1.67 | 2.41E-03 |
| *Il1rn* | 1.67 | 1.56E-02 |
| *Cfh* | 1.66 | 7.94E-03 |
| *Ilf3* | 1.65 | 4.11E-03 |
| *Hif1a* | 1.65 | 1.53E-02 |
| *Stat3* | 1.65 | 6.92E-03 |
| *Ccr5* | 1.64 | 1.45E-02 |
| *Ythdf2* | 1.64 | 1.87E-02 |
| *Mavs* | 1.64 | 1.96E-03 |
| *Cxcl16* | 1.64 | 2.59E-02 |
| *Nfatc3* | 1.63 | 1.01E-02 |
| *Stat6* | 1.62 | 2.40E-03 |
| *Atg5* | 1.62 | 1.34E-02 |
| *Map2k4* | 1.61 | 2.62E-04 |
| *Itga6* | 1.60 | 3.03E-02 |
| *Akt3* | 1.60 | 2.85E-02 |
| *Map3k7* | 1.60 | 6.69E-03 |
| *Lamp1* | 1.59 | 4.82E-03 |
| *Cd63* | 1.55 | 1.04E-02 |
| *Lyn* | 1.55 | 1.24E-02 |
| *Prkce* | 1.55 | 2.72E-02 |
| *Cmklr1* | 1.54 | 4.28E-02 |
| *Irak4* | 1.54 | 1.22E-02 |
| *Irf5* | 1.54 | 1.76E-02 |
| *Tgfb3* | 1.53 | 4.31E-02 |
| *Atg7* | 1.53 | 3.23E-02 |
| *Xbp1* | 1.53 | 1.49E-02 |
| *Ccnd3* | 1.52 | 9.76E-03 |
| *Psma2* | 1.52 | 4.67E-02 |
| *Tgfbr2* | 1.52 | 1.63E-02 |
| *Cd81* | 1.51 | 5.03E-03 |
